# Dual-Energy CT for Evaluation of Intra- and Extracapsular Silicone Implant Rupture

**DOI:** 10.1155/2016/6323709

**Published:** 2016-01-28

**Authors:** Katrina N. Glazebrook, Shuai Leng, Steven R. Jacobson, Cynthia M. McCollough

**Affiliations:** Mayo Clinic, 200 First Street SW, Rochester, MN 55904, USA

## Abstract

Silicone implants are commonly used for both breast augmentation and breast reconstruction. With aging of the implant, the silicone envelope may become weak or may rupture. The technique of choice for evaluation of implant integrity is breast MRI; however this may be contraindicated in some patients or the cost may be prohibitive. Dual-energy CT allows determination of density and atomic number of tissue and can provide material composition information. We present a case of extracapsular implant rupture with MRI and dual-energy CT imaging and surgical correlation.

## 1. Introduction

Many silicone gel implants placed in the 1990s may be ruptured by now. Robinson et al. performed Kaplan-Meier survival analyses which showed that the proportion of patients with intact implants after 20 years was as low as 5% [1, 2]. We describe the MRI and dual-energy CT (DECT) appearance of intra- and extracapsular rupture with nodal silicone deposits and peri-implant seroma in a patient with long-standing silicone implants following trauma.

## 2. Case Report

**Figure 1:**
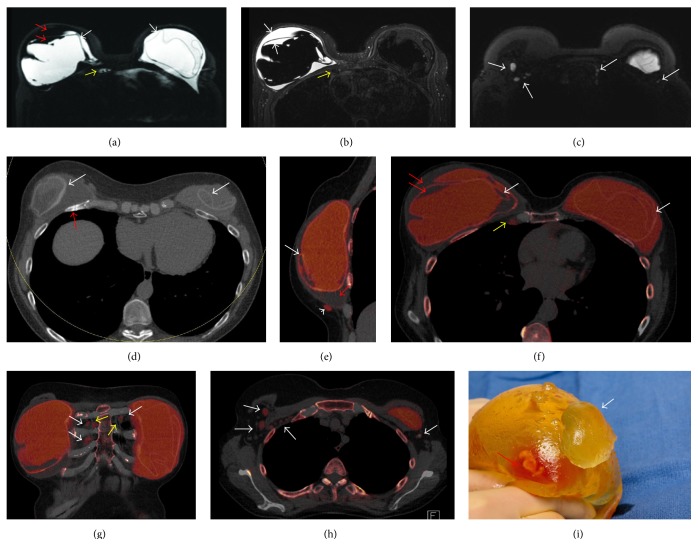
62-year-old woman presents with enlarging right breast following a fall one month earlier and a history of long standing bilateral subpectoral silicone implants following left modified radical mastectomy and simple right mastectomy in 1992. (a) Axial silicone sensitive MR sequence shows bilateral MR linguini sign of intracapsular rupture (white arrows) with low signal intensity material both within the capsule itself, along with high signal intensity silicone, and within the collapsed right envelope (red arrows). The high signal intensity tissue adjacent to the right internal mammary (IM) vessels (yellow arrow) was not appreciated as being likely due to silicone due to the inhomogeneity of the fat and water suppression about heart. (b) T2 weighted axial IDEAL MR sequence shows high T2 signal fluid of intracapsular seroma within the right envelope (white arrows). There is low signal intensity tissue adjacent to the right IM vessels which in retrospect would be in keeping with silicone within an internal mammary node (yellow arrow). (c) Axial silicone sensitive sequence from the high axilla demonstrates high signal intensity material which was not appreciated prospectively as right intranodal silicone in level I and II nodes and left IM nodes (arrows). (d) Axial mixed energy CT shows the CT linguini sign of collapsed implant envelopes bilaterally (white arrows). Healing fractured right anterior rib is clearly seen (red arrow). (e) Sagittal dual-energy noncontrast CT of the right breast with silicone colored as red shows intracapsular rupture with CT equivalent linguini sign of collapsed silicone envelope (white arrow). Water density material (red arrows) is noted surrounding the collapsed right envelope as seen on fluid sensitive MRI sequence (c). Extracapsular silicone is noted inferiorly (arrowhead). (f) Axial dual energy noncontrast CT of the right breast with silicone colored as red shows bilateral intracapsular rupture with CT equivalent linguini sign of collapsed silicone envelopes (white arrows). Water density material (red arrows) is noted surrounding and within the collapsed right envelope as seen on fluid sensitive MRI sequence (c). Silicone is also easily seen within enlarged right IM lymph node (yellow arrows). (g) Coronal DECT with color coding shows silicone within the right and left internal mammary nodes (white arrows). The internal mammary vessels can be seen (yellow arrows). (h) Axial DECT with silicone colored as red show silicone within right level I and II and left level I nodes (arrows). (i) Photograph of the explanted right implant with extruded silicone from the envelope (white arrow). Serosanguineous fluid noted within the envelope as seen on MRI and CT (red arrow).

A sixty-two-year-old woman was diagnosed with multicentric ductal carcinoma in situ and invasive ductal carcinoma of the left breast in 1992. She underwent a left modified radical mastectomy and a right simple mastectomy with immediate bilateral reconstruction with subpectoral silicone gel implants. A month before presentation, she fell on the right breast, heard a pop, and had immediate right-sided chest wall pain. She noted progressive enlargement of the right breast with increasing pain. Clinically, the right breast was larger than the left, suspicious for seroma and or hematoma with bilateral animation deformity consistent with subpectoral implants. An MRI exam was performed to evaluate implant rupture using an 8-channel breast coil. The MRI exam (1.5 T Signa LX Echospeed, General Electric Medical Systems, Milwaukee, WI) consisted of an axial T2 weighted IDEAL sequence, axial and sagittal silicone sensitive series, and pre- and postcontrast Vibrant 3D T1 weighted gradient series. Intracapsular rupture was found on the right with a surrounding seroma, both within the ruptured envelope and mixing with the silicone outside the envelope but within the fibrous capsule (Figures 1(a) and 1(b)). Foci of extracapsular silicone were felt to be present in the right axillary tail. Intracapsular rupture of the left implant was also noted with MRI linguini sign [3] (Figures 1(a) and 1(b)). No axillary adenopathy was identified as the high signal intensity on the silicone sensitive sequence was not appreciated prospectively to correspond to silicone within level I and II axillary nodes (Figure 1(c)). There was no abnormal enhancement to suggest tumor recurrence.

DECT was performed using a dual-source CT scanner (SOMATOM Force, Siemens Healthcare, Forchheim, Germany) using tube potentials of 100 and 150 kV. An additional tin filter was added to the 150 kV beam to increase spectral separation. The patient was scanned prone with a single acquisition using a prototype breast stand modified from a 16-channel breast MRI coil for CT use. No intravenous contrast material was given. Tube current was adjusted to match the radiation dose as that of a routine noncontrast chest CT. The volume CTDI (CTDI_vol_) was 7.19 mGy, and the dose length product (DLP) was 216.8 mGy·cm. Axial images were reconstructed with slice thickness of 1.5 mm. Sagittal and coronal reformats were performed. Images were analyzed using the 3-material decomposition algorithm of the dual-energy CT software (syngo Via Dual Energy, Siemens Healthcare) with “Liver VNC” workflow. The mixed CT images (average if 90 and 150 kV images) demonstrated healing nondisplaced rib fractures of the right 3rd through 6th ribs and left 3rd through 5th ribs anterolaterally (Figure 1(d)). On the silicone color-coded images of the right breast, fluid density material was noted within the right fibrous capsule external to and within the collapsed silicone envelope consistent with seroma and intracapsular rupture (Figure 1(e)). Several foci of extracapsular silicone were noted superficial to the pectoralis muscle in the inferomedial breast that was not appreciated prospectively on the breast MRI exam (Figure 1(e)). Intracapsular rupture was also seen on the left, with fluid noted within the collapsed envelope (Figure 1(f)). No extracapsular silicone was seen in the left breast. There were several enlarged internal mammary nodes bilaterally containing silicone (Figures 1(f) and 1(g)) and level I through III (right) and level I (left) axillary nodes containing silicone (Figure 1(h)). Note was also made of coronary artery calcifications. This led to a cardiac stress test, which the patient failed, and resulted in 3-vessel coronary artery stent placement with good results.

The patient underwent explantation of both implants with bilateral capsulectomies and silicone implant exchange with AlloDerm placement. The explanted right implant showed signs of rupture with seroma actually within the implant itself, as shown on MRI and DECT (Figure 1(i)). Rupture of the left implant was obvious following entry of the capsule.

On follow-up, the patient was very satisfied with her new implants and very grateful for the DECT for identifying the coronary calcifications.

## 3. Discussion

The prevalence of silicone breast implant rupture in a population-based study has been reported to be as high as 55%, with 22% of ruptured implants showing extracapsular spread of silicone [4, 5]. Local complications and adverse outcomes include capsular contracture, reoperation, and removal. Women may also experience breast pain, wrinkling, asymmetry, scarring, and rarely infection [5]. Breast ultrasound has been used to evaluate implant integrity with sensitivity and specificity ranging from 50 to 77% and from 55 to 84%, respectively [3, 6–9]. The sonographic sign of intracapsular rupture is the “stepladder” sign, with multiple curvilinear reflective lines within the interior of the implant corresponding to the collapsed envelope. Silicone masses within the breast tissue and axillary nodes in cases of extracapsular rupture have a typical “snowstorm” appearance with posterior shadowing [3, 6–9].

Breast MRI is the technique of choice for assessment of implant integrity. The reported sensitivity and specificity for implant rupture range from 74 to 100% and from 55 to 84%, respectively [5, 8–11]. Intracapsular rupture of the implant envelope is seen as hypodense linear lines lying within the hyperintense silicone, the so-called “Linguini” sign [3]. Silicone-specific sequences with 3-point chemical shift techniques show high signal intensity silicone within the breast parenchyma or axillary nodes in extracapsular rupture. The FDA has mandated a surveillance MRI screening examination for silent rupture in patients at 3 years following implantation of silicone breast implants and every 2 years thereafter [4]. MRI may not be possible in patients with contraindications to MRI (e.g., pacemaker, cochlear implant) or claustrophobia. The costs of MRI surveillance are not typically covered by insurance and can be prohibitively expensive for most patients.

Johnson et al. performed DECT for the evaluation of silicone breast implants [12]. Seven silicone implant specimens were evaluated with DECT using 100 and 140 kV tube potentials, with a strong dual-energy signal. Two patients scheduled for implant removal or replacements were examined. In one patient, both implants were intact, while rupture was identified in the other patient. Ultrasound, MRI, surgical findings, and histology confirmed the DECT diagnosis.

CT number (in Hounsfield units) depends on X-ray attenuation, which depends on the physical density (g/cm^3^) (electron-density) and atomic number (*Z*). Different materials may have the same CT number if atomic number differences are offset by density differences [13]. DECT allows determination of density and *Z* and can provide material composition information. Silicone contains the metalloid element silicon with element number 14. Soft tissue contains lighter elements such as hydrogen and oxygen. Silicon and soft tissue have different slopes in a plot of low-energy CT number versus high-energy CT number and therefore can be differentiated. This technique has been shown to be highly accurate in the characterization of kidney stones and identification of monosodium urate crystals in the extremities in patients with gout [14–17]. In this case, DECT not only defined the intracapsular ruptures and right seroma, but also more clearly identified the extracapsular silicone within chest wall tissue, level I through III axillary nodes, and extra-axillary nodes, compared to the MRI scan. Additional clinically relevant information was also noted on the DECT, including identification of the bilateral rib fractures and coronary artery calcifications.

## 4. Conclusion

While MRI is the current technique of choice for evaluation of intra- and extracapsular silicone breast implant ruptures, DECT however shows promise in more specifically evaluating the extent of extracapsular rupture and nodal involvement in a single, noncontrast, breathhold scan.
